# Carotid artery dissection in Hutchinson-Gilford Progeria: a case report

**DOI:** 10.1186/s12887-022-03179-4

**Published:** 2022-03-14

**Authors:** Víctor González-Maestro, Einés Monteagudo-Vilavedra, Jorge Rodríguez-Antuña, Marta Lendoiro-Fuentes, María Soledad Brage Gómez, Elena Maside Miño

**Affiliations:** 1 Radiology Department, Complexo Hospitalario Universitario de Ferrol, Sergas, Spain; 2 Paediatrics Department, Complexo Hospitalario Universitario de Ferrol, Sergas, Spain

**Keywords:** Carotid, Dissection, Stroke, Hutchinson-Gilford´s Progeria

## Abstract

**Background:**

Strokes in the paediatric age group have their own epidemiology and aetiology and are frequently misdiagnosed. As in the adult population, they present some risk factors that must be identified. Cerebral arteriopathies as a cause of paediatric ischaemic stroke present a very diverse aetiology and morphology.

In this article we report a paediatric stroke in a patient who was diagnosed during his first months of life of Hutchinson-Gilford´s Progeria (HGP). This is a rare genetic condition caused by mutations in the LMNA gene, producing an aberrant lamin A protein. The disease leads to premature aging, and cardiovascular complications are the first cause of morbidity and mortality in these patients.

**Case presentation:**

We report the case of a 5-year-old patient with HGP (missense mutation—de novo—c.1822G > A in heterozygosis, LMNA gene). The patient was diagnosed during his first year of life and presented distinct phenotypical features. No other relevant comorbidities were present.

He was admitted to the emergency department for right hemiparesis with at least 4 h of evolution, with inability to open the hand and slight decrease in the level of consciousness (pedNIHSS 5–6). Cranial-CT and angio-CT showed findings indicative of left carotid dissection. Consensus was reached on conservative medical management with anticoagulation and antiplatelet therapy. In the first few days, the patient had a favourable evolution with resolution of the right lower limb hemiparesis and, one month after discharge, of the hand paresis.

**Conclusions:**

The clinical manifestations, the vascular phenotype of the genetic mutation and the location of the radiological signs on a specific vascular morphology are indicative of carotid dissection. Spontaneous dissections occur under a predisposing risk factor or disease and are an exceptional finding in patients with HGP.

**Supplementary Information:**

The online version contains supplementary material available at 10.1186/s12887-022-03179-4.

## Background

Strokes in the paediatric age group have their own epidemiology and aetiology and are frequently misdiagnosed or their diagnosis is delayed. In addition, as in the adult population, they present some underlying risk factors that must be identified. With an estimated incidence of 1:4 million births, there are 132 cases of Hutchinson-Gilford´s Progeria (HGP) [1] whose morbidity and mortality are related to cerebrovascular events; but to date and to our knowledge, no case of arterial dissection has been described.

HGP is a rare genetic condition caused by mutations in the lamin A protein producing progerin, an aberrant protein. Most patients present de novo heterozygous dominant mutations in the LMNA gene. The disease leads to premature aging and cardiovascular complications are the first cause of morbidity and mortality in these patients [2, 3].

## Case presentation

The patient is a 5-year-old male diagnosed in the first months of life with HGP disease. He was born at 34 + 6 weeks of gestational age but during neonatal period he did not present any pathological feature. Around two months of age, dermatological abnormalities consisting of sclerotic skin and alopecia appeared. A skin biopsy was performed, histopathological findings suggested a progeroid syndrome. The genetic study found a pathogenic mutation in LMNA gene (missense mutation—de novo—c.1822G > A in heterozygosis), as previously reported and described.

He developed phenotypical characteristics of HGP with micrognathia, prominent forehead and thin lips, loss of subcutaneous fat tissue and prominent abdomen, among others. During follow-up, at one year of life he was diagnosed with mild hypertrophic cardiomyopathy with asymmetric septal hypertrophy, currently asymptomatic. He has subclinical hypothyroidism, and his neurological development is in accordance with his chronological age. He receives Levothyroxine and Fluticasone.

The patient was admitted to the Emergency Department for right hemiparesis of at least 4 h' evolution. Physical examination confirmed limitation to abduction of the right arm without counter-resistance. The right hand shows flexed fingers without opening and the leg claudicates in less than 5 s. Together with a slight decrease in the level of consciousness, these data would configure a pedNIHSS 5–6. The remaining neurological examination and vital signs are normal.

Less than 1 h after arrival, a cranial-CT scan was performed which showed no ischaemic signs (Online resource 1) and an angio-CT scan from the neck to the vertex, after conscious sedation with iv Propofol and Iohexol 350 mg l/ml adjusted for age and weight as the contrast medium.

**Fig. 1:**
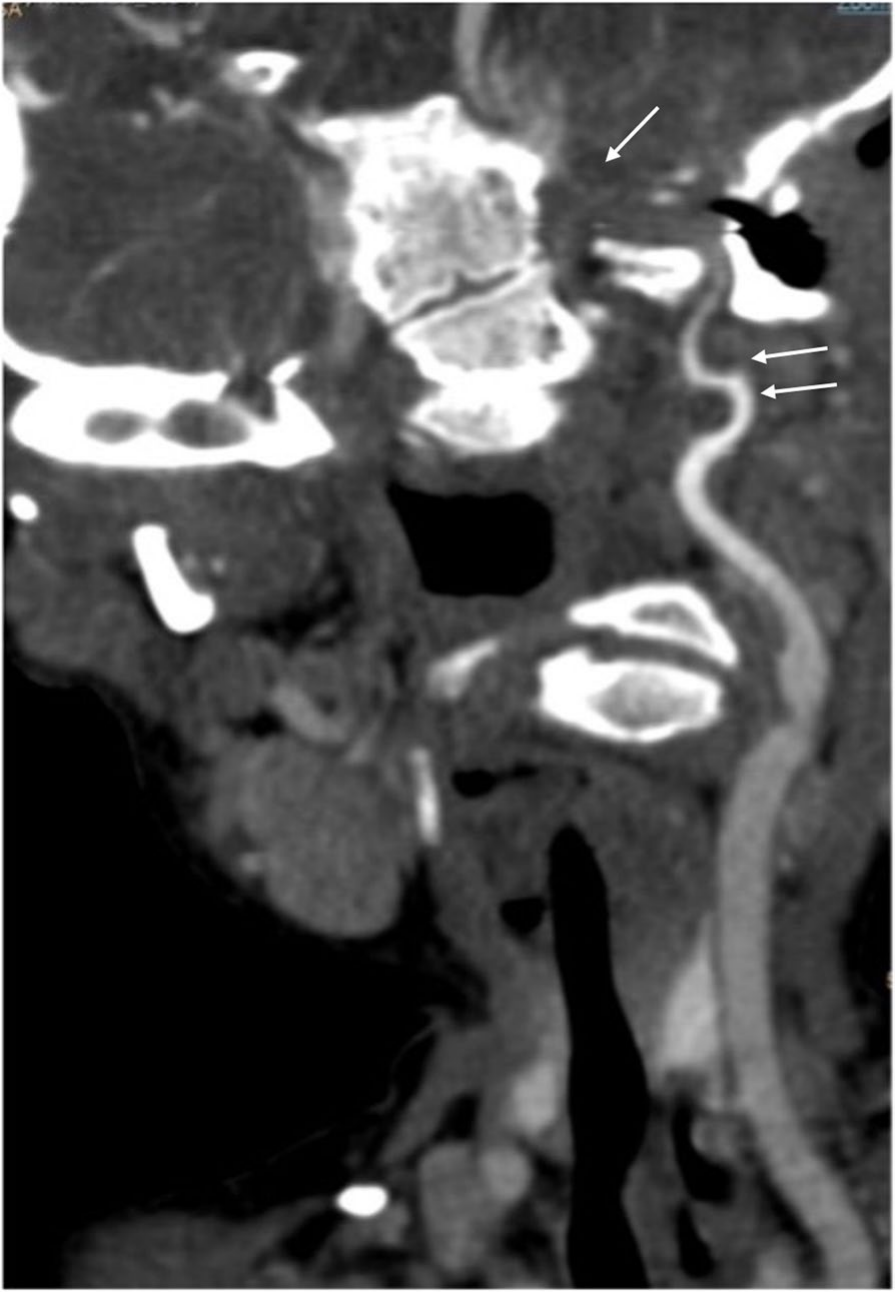
Occlusion of the internal carotid artery in the petrous segment to the cavernous segment (single arrow) preceded by a long and progressive stenosis in the sub-petrous segment (double arrow)

**Fig. 2:**
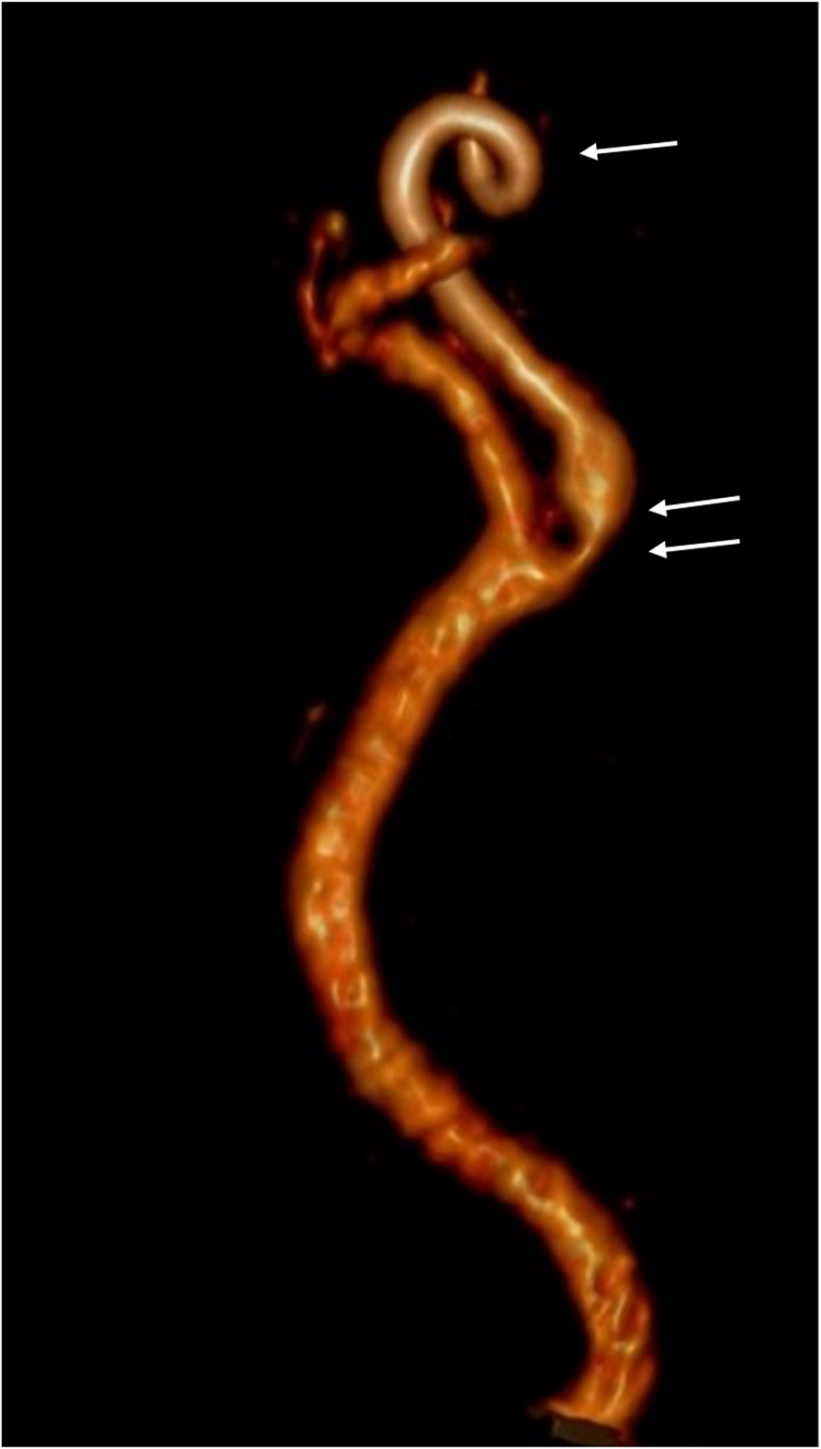
Coiling in sub-petrous segment (single arrow).Supra bulbar stenosis and post-stenotic dilatation (double arrow)

An occlusion is identified in the petrous segment up to the cavernous segment of the left internal carotid artery preceded by a pre-occlusive stenosis in the sub-petrous segment with higher contrast density (Fig. 1) describing a coiling morphology. Immediately superior to the carotid bulb, a significant stenosis is observed followed by post-stenotic dilatation (Fig. 2). No intracranial thrombus or significant atheromatous plaques are identified. (Online resource 2).

The overall radiological findings are indicative of carotid artery dissection without associated intracranial thrombus.

The patient was admitted from the Emergency Department and conservative medical treatment with Enoxaparin (dose 1 mg/kg/12 h) and acetylsalicylic acid (5 mg/kg/day) was agreed. Complete blood count, biochemical profile and a basic coagulation study showed no relevant findings. Minimal levels of anti-Xa factor (0.18) were found.

He progressed favorably, with no additional neurological deficits indicative of a new stroke. In the first hours of admission, he recovered mobility of the lower limb, allowing him to stand upright.

Recovery of the right upper limb was more progressive, with resolution of the hand paresis approximately one month after admission. Currently, acetylsalicylic acid is maintained at an antiplatelet dose (40 mg/24 h). At an external center, 2 months after the neurological event, treatment was started on a compassionate use basis with oral Lorafenib.

## Discussion and conclusions

Cervical arterial dissection is one of the possible aetiologies of paediatric stroke. Ruling out traumatic origin, spontaneous arterial dissection occurs in certain patients with pathologies or risk factors that favour vascular wall injury [4]. In this regard, the mutation underlying HGPS renders the structure and function of the vascular wall defective [5]. The finding of arterial dissection has not been described to our knowledge. A vasculopathy unique to HGPS has been proposed as a cause of stroke since the hypothesis of atheromatosis has not been validated [6], as in our case. (Online resource 2 and 3).

Our case is a long occlusion from the petrous segment (typical location of carotid dissections [7]) to the cavernous segment. It is preceded by a long and progressive stenosis in the sub-petrous segment (long tapering stenosis) which describes a typical course of arterial coiling, a sign of severe arterial tortuosity, recognized as a risk factor for the development of dissection [8].

The clinical manifestations [9], vascular phenotype of the mutation, location and radiological signs described on a prone vascular morphology are indicative of carotid dissection. Our limitations for this case are clear. Inherent to the patient, the difficulty in managing conscious sedation [10] prior to performing imaging tests. In addition, local technical limitation to the use of urgent MRI results in a lower accuracy and diagnostic capacity of the mural hematoma due to dissection and cerebral ischaemic changes in the acute phase. However, angio-CT as well as angio-MRI [11] seem to have similar accuracy in the diagnosis of occlusion and the described long previous stenosis.

On the other hand, the therapeutic management of paediatric stroke remains a challenge, with poor consensus and low scientific evidence. It must always be adapted to the particularities of the case, exhaustively assessing the presence of risk factors and the possibility of intervention. Since it is an extracranial dissection, medical treatment is started. After six days, anticoagulation is suspended due to the erratic absorption of the patient (minimum values of factor Xa) and the current controversy surrounding medical treatment of carotid dissection [12].

In conclusion, arterial dissection is one of the cerebral arteriopathies causing stroke in childhood. Ruling out the traumatic event, spontaneous dissections occur under a predisposing risk factor or disease and are an exceptional finding in pediatric patients and particularly in patients with HGP.

## Supplementary Information

**Additional file 1. **Laboratory tests.

**Additional file 2: Online resource 1.** No tomographic signs of acute ischemia are identified, but in the right centrum semiovale there is a hypodense area which probably reflects a chronic silent stroke typical in this kind of patients.

**Additional file 3: Online resource 2.** Left internal carotid occlusion in petrous segment, preceded by critical stenosis with coiling morphology in sub-petrous segment. Hypertrophy of anterior and posterior spinal arteries.

**Additional file 4: Online resource 3.** Occlusion of left internal carotid artery in petrous segment, preceded by critical stenosis with coiling morphology in sub-petrous segment. Short stenosis in cavernous segment of right carotid artery and at junction of segments V2-V3 of left vertebral artery.

## Data Availability

The data that support the findings of this study are available from SERGAS-Consellería de Sanidade, but restrictions apply to the availability of these data, which were used under license for the current study, and so are not publicly available. Data are however available from the authors upon reasonable request.

## Abbreviations

HGP: Hutchinson-Gilford's Progeria
pedNIHSS: Pediatric National Institutes of Health Stroke Scale
Cranial-CT: Cranial computered tomography
Angio-CT: Angiography computered tomography
Angio-MRI: Angiography magnetic resonance imaging
