# Supplementary material for: Carotid artery dissection in Hutchinson-Gilford Progeria: a case report
Source: BMC Pediatr. 2022 Mar 14;22:135. doi: 10.1186/s12887-022-03179-4 (PMC8922814; doi:10.1186/s12887-022-03179-4)
Supplement: Supplementary file 1 — Additional file 1. Laboratory tests. [file 12887_2022_3179_MOESM1_ESM.pdf]

LABORATORY TESTS ON ADMISSION

**Complete blood count (CBC)**	
Haemoglobin	13.9 g/dL
Haematocrit	39.2%
MCV	72.0 fL
Platelets	359.000/µL
White blood cells	10.180/µL
Neutrophils	4.700/µL
Lymphocytes	4.000/µL
Monocytes	800/µL

**Biochemical profile**	
Glucose	102 mg/dL
Urea	19 mg/dL
Creatinine	0.13 mg/dL
GPT	18 U/L
LDH	211 U/L
CK	68 U/L
Sodium	137 mEq/L
Potassium	4.2 mEq/L
C Reactive Protein	2.17 mg/L
High-sensitivity troponin	3 ng/L

**Coagulation study**	
PT	12.0 seg
PT control	11.7 seg
PT ratio	1.03
aPTT	32.8 seg
aPTT control	31.8 seg
aPTT ratio	1.03
INR	1.04
